# Lurasidone in the Treatment of Bipolar Depression: Systematic Review of Systematic Reviews

**DOI:** 10.1155/2017/3084859

**Published:** 2017-05-09

**Authors:** Michele Fornaro, Domenico De Berardis, Giampaolo Perna, Marco Solmi, Nicola Veronese, Laura Orsolini, Elisabetta Filomena Buonaguro, Felice Iasevoli, Cristiano André Köhler, André Ferrer Carvalho, Andrea de Bartolomeis

**Affiliations:** ^1^Laboratory of Molecular and Translational Psychiatry, Department of Neuroscience, School of Medicine, University “Federico II”, Naples, Italy; ^2^New York State Psychiatric Institute, Columbia University, New York, NY, USA; ^3^National Health Service, Department of Mental Health, Psychiatric Service of Diagnosis and Treatment, Hospital “G. Mazzini”, ASL 4, Teramo, Italy; ^4^Department of Clinical Neurosciences, Hermanas Hospitalarias, Villa San Benedetto Menni Hospital, FoRiPsi, Albese con Cassano, 22032 Como, Italy; ^5^Department of Psychiatry and Neuropsychology, Maastricht University, 6200 MD Maastricht, Netherlands; ^6^Department of Psychiatry and Behavioral Sciences, Leonard Miller School of Medicine, Miami University, Miami, FL 33136, USA; ^7^Department of Neurosciences, University of Padua, Padua, Italy; ^8^Institute for Clinical Research and Education in Medicine (IREM), Padua, Italy; ^9^Department of Medicine (DIMED), University of Padua, Padua, Italy; ^10^School of Life and Medical Sciences, University of Hertfordshire, Hatfield, Herts, UK; ^11^Translational Psychiatry Research Group and Department of Clinical Medicine, Faculty of Medicine, Federal University of Ceará, Fortaleza, CE, Brazil

## Abstract

**Introduction:**

A burgeoning number of systematic reviews considering lurasidone in the treatment of bipolar depression have occurred since its Food and Drug Administration extended approval in 2013. While a paucity of available quantitative evidence still precludes preliminary meta-analysis on the matter, the present quality assessment of systematic review of systematic reviews, nonetheless, aims at highlighting current essential information on the topic.

**Methods:**

Both published and unpublished systematic reviews about lurasidone mono- or adjunctive therapy in the treatment of bipolar depression were searched by two independent authors inquiring PubMed/Cochrane/Embase/Scopus from inception until October 2016.

**Results:**

Twelve included systematic reviews were of moderate-to-high quality and consistent in covering the handful of RCTs available to date, suggesting the promising efficacy, safety, and tolerability profile of lurasidone. Concordance on the drug profile seems to be corroborated by a steadily increasing number of convergent qualitative reports on the matter.

**Limitations:**

Publication, sponsorship, language, citation, and measurement biases.

**Conclusions:**

Despite being preliminary in nature, this overview stipulates the effectiveness of lurasidone in the acute treatment of Type I bipolar depression overall. As outlined by most of the reviewed evidence, recommendations for future research should include further controlled trials of extended duration.

## 1. Introduction

Although the Diagnostic and Statistical Manual for Mental Disorders-Fourth Edition (DSM-IV) [1] poses mania as the hallmark of bipolar disorder (BD), depression is often the most enduring facet of the illness as emphasized by the Fifth edition of the Manual [2] requiring significant treatment efforts [3]. Yet, only a handful of randomized clinical trials (RCTs) exist about bipolar depression in comparison to major depressive disorder (MDD), which may be because unipolar “endogenous” or “melancholic” major depression episodes (MDEs) have long been considered equivalent to bipolar MDEs, from clinical, neurobiological, and treatment-modality standpoints [4]. Regrettably, the only reservation was that antidepressants might switch to the manic pole; thus the enduring common clinical practice among clinicians was to seamlessly transpose the clinical data and wisdom from the treatment of unipolar to bipolar depression [5].

This later misconception has long been corroborated by anecdotal reports suggesting that most clinicians may still perceive medications as belonging to a class with regard to a specific therapeutic action rather than based on aimed “neuroscience-nomenclature” approach grounded on the pharmacological profile of the drug [6], both in the specialty and in general practice settings accessed by patients with BD [7]. Yet, therapeutic “class effect” in BD is an exception rather than rule [8].

The majority of patients with bipolar depression fail to respond adequately to pharmacotherapy [9], whereas the use of standard antidepressant medications, even when associated with established mood-stabilizers, poses major efficacy concerns beyond overall short- and long-term tolerability issues, especially for Type I BD (BD-I) and/or in the presence of associated mixed and/or atypical features, just to mention few [10, 11].

In contrast, evidence in support of the use of at least some of the second-generation antipsychotic (SGA) mono- or add-on therapy either for MDD [12, 13] or bipolar depression [14–18] is increasing over the time, though additional safe and effective Food and Drug Administration- (FDA-) approved SGAs for bipolar depression are solicited [19].

Currently, olanzapine-fluoxetine combination (OFC), quetiapine (either the standard or the extended release preparation), and lurasidone are the only FDA drugs granted (extended) approval for the (acute) treatment of bipolar depression in adults [20, 21].

Lurasidone received FDA approval for the treatment of schizophrenia in adults in October 2010 and was granted extended approval on June 2013 for the treatment of acute depression associated with BD-I in adults [22], either as monotherapy [23] or as adjunctive treatment to either lithium or valproate [24] flexible-dose regimen trials, further assessed in subsequent retrospective/prospective data analysis [25].

Potential* safety* (namely, the risk to the subject/patient, usually assessed in by laboratory testing [e.g., clinical chemistry and hematology], physical examination [vital signs], clinical adverse event[s], AEs, and other tests, e.g., the electrocardiogram),* tolerability *(namely, the degree to which overt AEs can be tolerated by the subject/patient), and* effectiveness* (usually, “effectiveness” trials [pragmatic trials] measure the degree of beneficial effect under “real world” clinical settings rather than the “efficacy” tested by “explanatory” trials aiming at determining whether an intervention produces the expected result under ideal circumstances) of adjunctive lurasidone across a broad range of treatment resistant BD outpatients otherwise excluded by routine clinical trials (e.g., those with mixed and/or rapid-cycling features; those taking additional psychiatric or nonpsychiatric medications; and/or older-age cases of BD) have been preliminarily postulated [26–29] though critically appraised by independent authors [30].

While the overall effect size of OFC, quetiapine (regardless of release formulation), and lurasidone in mitigating depressive symptoms is similar, the later one showed a lower propensity for weight gain as well as overall metabolic neutrality in the bipolar population [20, 21].

Lurasidone, compared to previous FDA-approved SGAs for bipolar depression, yielded comparable benefits (all had single-digit number needed [NNT] for treatment versus placebo response or remission) and less risk of harm (double-digit or greater numbers needed to harm [NNH] with lurasidone compared to single-digit NNHs for sedation with quetiapine and for ≥7% weight gain with olanzapine-fluoxetine combination) and thus a substantially more favorable likelihood to be helped or harmed [LHH] (> or ≫1) with lurasidone monotherapy and adjunctive therapy, compared to quetiapine and olanzapine-fluoxetine combination (LHH < or ~1) [31]. NNT, NNH, and LHH represent widely well-recognized indexes most clinicians are progressively becoming familiar with [32]. Nonetheless, the overall clinical validity and generalizability of these later indexes have been seldom questioned by some [33].

A burgeoning number of systematic reviews have nonetheless occurred since the pivotal RCTs leading to FDA extended approval of lurasidone, as the need for better efficacy/tolerability profile drugs for bipolar depression still represents a priority for the prescribing clinicians, policy-makers, and the suffering ones, indeed.

More recently, calls have been made for updated brief reviews to provide decision-makers with the essential evidence they need in a shorter time frame, but the possible limitations of such brief reviews, compared to full-systematic reviews, require further methodological research [34].

In recent years, however, decision-makers who were once overwhelmed by the number of individual studies have become faced by a plethora of reviews [35, 36]. This is compelling, especially when novel compounds of potential priority interest for the clinical practice like lurasidone, even in the treatment of BD, may need to await for additional RCTs to allow reliable meta-analytic pooling [37, 38].

Therefore, while awaiting for additional primary research trials to allow reliable and large-scale quantitative extractions, varying approaches have been proposed [39] to promote quality assessment of systematic reviews of systematic reviews (or overviews) to be eventually accounted for subsequent umbrella reviews (broad coverage of systematic reviews and meta-analyses) [40]. Among others, several approaches including the Preferred Reporting Items for Systematic review and Meta-Analysis-Protocols, PRISMA-P [41] (formerly, PRISMA, QUOROM), or organizations devoted to the preparation of systematic reviews, including the National Institute of Health and Clinical Excellence (NICE) in the UK, the Evidence-based Practice Centre Program in the US, the Joanna Briggs Institute, and the International Campbell and Cochrane Collaborations arose [39]. It was not until 2009 that “A Measurement Tool to Assess Systematic Reviews” (AMSTAR) was documented [42], proving to be a potentially reliable and valid assessment tool to specifically evaluate the methodological quality of systematic reviews [43].

Therefore, the aim of the present overview was to assess the methodological quality of systematic reviews assessing the evidence about lurasidone in the treatment of bipolar depression, with the ultimate goals of (i) ranking and prioritizing those reviews for which the methodology would allow reliable conclusions and recommendations for the future needs; (ii) critically pointing out which unmet needs and expectations have been highlighted by the clinician authors to be implemented by future lines of research about the pharmacological treatment of acute bipolar depression in adults with a special emphasis towards lurasidone, prompting for attention by policy and clinical decision-makers.

## 2. Materials and Procedures

Overall methods and procedures resemble those adopted by recent peer-review articles involving the use of AMSTAR methodology [42, 43], as applied to heterogeneous medical fields of research [45].

### 2.1. Search Strategy for the Identification of Systematic Reviews

A protocol was drafted before the implementation of the review (a copy is available from the authors). Searches were conducted of Medline (via PubMed), EMBASE and the Cochrane library (which includes the DARE database of abstracts of reviews on interventions), and Scopus, on October 14, 2016, and included a combination of free text and MeSH terms (please refer to Table 1). Despite the relatively recent introduction of lurasidone, indexes were searched from inception aiming at gathering as much information as possible on the topic. Searches were limited to “*systematic reviews*” or “*review*” type publication across varying sources. No language restrictions were imposed although only English language results were retained. This strategy was purportedly performed to get a better insight about any eventual language and regional publication differential trend on the topic. The* title and abstract* of each article were scanned (independently by two reviewers: MF and LO) and full-reprints of articles of potentially eligible reviews were obtained. Potentially eligible reviews were then screened, again independently by two reviewers, per the review selection criteria outlined below. All resulting references were further screened for identification of additional reviews.

### 2.2. Review Selection Criteria

Only systematic reviews were included. Case reports, controlled RCT (which were not part of a review), were excluded. Those narrative reviews indexed by major databases as “systematic” were nonetheless identified and then anyway screened for eventual inclusion and overall assessment. Both systematic reviews of RCTs and observational studies were eligible for inclusion. To be considered for inclusion, the review had to include evidence about the use of lurasidone (any dose) for bipolar disorder (any mood polarity), either for the acute or for the maintenance mono- or adjunctive treatment therapy. Systematic reviews covering drugs other than lurasidone or any additional non-BD prescription of lurasidone were likewise included in the present overview. Populations at interest were therefore “*BD patients exposed to lurasidone.*”

### 2.3. Preliminary Data Abstraction

For each review meeting the inclusion criteria data were abstracted independently by two reviewers (MF and DDB). All data was compared and identified anomalies rectified by mutual agreement. Data were obtained exclusively from the systematic reviews, while additional manual screening was planned to enrich the search strategy. The primary study reports were likewise reviewed before assessment of systematic review reporting on the matter. Data abstracted from each systematic review included (i) authors and date of publication; country of origin (leading author most current affiliation); major biases, including sponsorship bias. In case of reviews covering multiple RCT studies, these later ones were punctually referenced in Table 2 (please see the text for results).

### 2.4. Assessment of Data Quality

The methodological quality and risk of bias of the systematic reviews at interest were performed using the AMSTAR items [42, 43] by two independent reviewers (MF and DDB). Risk of bias of primary studies was not assessed where this was covered by the corresponding systematic reviews. However, we did not record whether and how the reviews had assessed the quality of the primary studies and which method had been used (for example the Cochrane risk of bias tool) [54]. Briefly, the AMSTAR is a tool aiming at addressing relevant domains (as applicable): establishing the research question and inclusion criteria before the conduct of the review, data extraction by at least two independent data extractors, comprehensive literature review with searching of at least two databases, key word identification, expert consultation and limits as necessary, detailed list of included/excluded studies and consideration of quality assessments in analysis and conclusions, appropriate assessment of homogeneity, assessment of publication bias, and a statement of any conflict of interest. Therefore, (i) the extent of searching undertaken; (ii) description of review selection and inclusion criteria; (iii) assessment of publication bias; (iv) assessment of heterogeneity; and (v) comparability of included reviews are emphasized by the AMSTAR method [39]. The AMSTAR checklist items are presented in the form of questions, with possible responses of “yes” (item/question fully addressed), “no” (item/question not addressed), “cannot answer” (not enough information to answer the question), and “not applicable,” https://amstar.ca/Amstar_Checklist.php; the actual eleven domains rated by the AMSTAR and its psychometric features have been further detailed elsewhere [43].

The revised assessment of multiple systematic reviews (R-AMSTAR) based on appendix 1 included in the open-access work by Kung et al. [44] was nonetheless integrated [55] to the standard AMSTAR to generate quantitative scores of qualitative evidence [44] since use of the original AMSTAR checklist alone would have posed subjectivity concerns and possibly reduced the reliability and replicability of assessments [56]. Specifically, stating the discrepancy existing about standardized scoring of the original AMSTAR domains [55–57], grading of the R-AMSTAR could vary across different authors and research themes [55]; we integrated two alternative scores methods in the assessment of each systematic review: (a) each of the individual eleven domains of the original instrument ranged between 1 and 4 (maximum); thus the R-AMSTAR total scores ranged between 11 (minimum) and 44 (maximum). Scoring for each domain could be determined based on the number of satisfied criteria varying across different domains rather than nonstandardized cut-off scores [44]; (b) based on the later scores values, a percentile ranking is obtained, for each individual score, which is then translatable into a letter grade based on the extent of PICO/PIPO research questions (population, intervention, comparison, prediction, and outcome) answered, with the following letters: A (best)–E (worst) range (variable across varying questions). Details about the domains and proposed scoring of the R-AMSTAR available in the public domain based on the appendix 1 are included in the work by Kung and colleagues [44, 55].

### 2.5. Exclusion of Duplicate Primary Studies (Document in Systematic Reviews)

Both auto- and hand-searches for “type I” (“duplicates among/across different databases”) and “type II” (“duplicate publications in different Journals/issues”) [58] were performed based on Thompson Endnote X7® for Microsoft Windows®.

Reviews were then screened to exclude systematic reviews with duplicate primary studies unless they reported on different outcomes or provided alternative critical account of the evidence.

### 2.6. Outcomes

Principal outcomes related to the impressions and recommendations about efficacy and tolerability made by the authors given a systematic review at review beyond the raw data come from the covered original RCTs. Specifically, overall conclusions drawn by the authors and unmet needs to be addressed by future studies were included providing a concise narrative synthesis based on each systematic review included in the present overview. Conversely, specific outcomes of treatment interventions reported by the original RCTs covered across varying reviews were not accounted herein. On the other side, it is worth mentioning that higher R-AMSTAR scores and letter grades (e.g., “A”) would indicate more reliable and trustworthy conclusions.

## 3. Results

**Figure 1:**
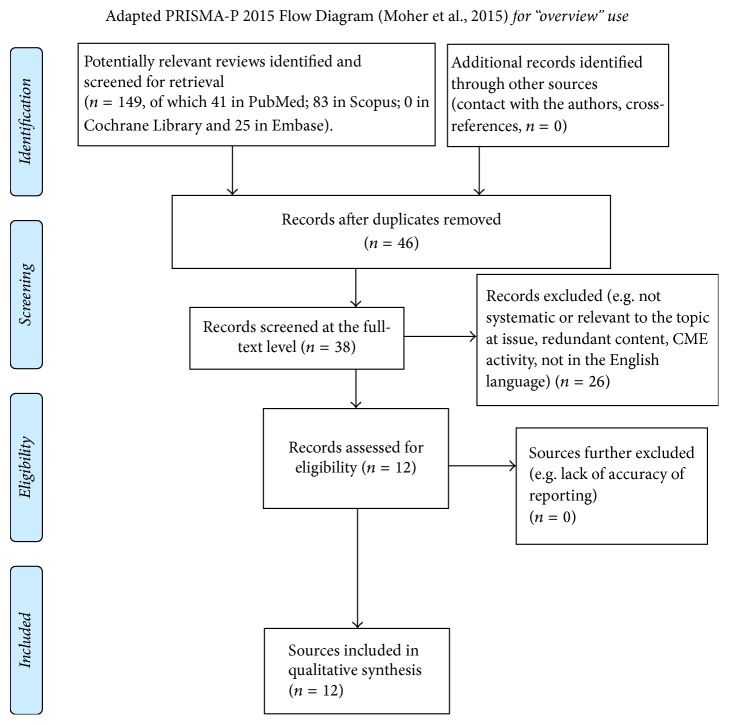
Flow chart of overview procedures.

Stating the lack of quantitative data to abstract (please refer to Figure 1 for study flow chart) (e.g., treatment effect estimates), overall synthesis of results could be presented in a narrative fashion (as outlined in Table 2).

### 3.1. Overview of the Systematic Evidence

**Figure 2:**
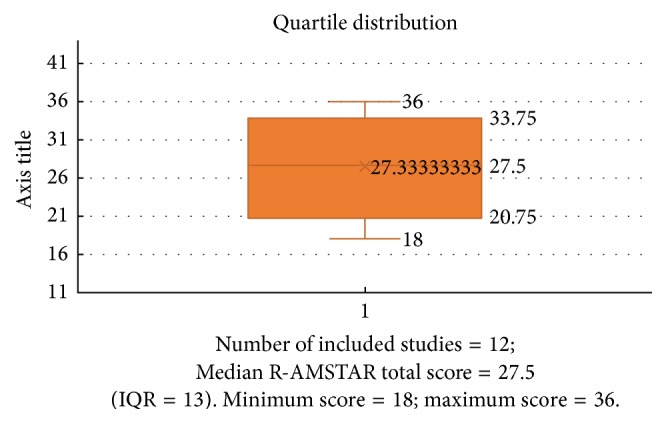
Companion box-plot and quartile distribution for Table 2: Conversion of raw R-AMSTAR total scores into percentile scores (see Table 3).

As depicted in Figure 2 and Table 3, most of the included “systematic” reviews fell in the upper tier of the quality range according to the percentile score (or related grade letter): R-AMSTAR total median score = 27.5 (Interquartile-Range [IQR] = 13), whereas the range of raw R-AMSTAR scores was equal to 18–36.

On average, only few original RCTs about lurasidone in the treatment of BD in adults could be “systematically reviewed” since 2013 (FDA extension of approval beyond schizophrenia). Moreover, both the pivotal acute mono- [23] and adjunctive [24] therapy trials were sponsored ones, as is preliminary data about maintenance [29, 30]. Additional details about study design, outcomes and measures of the RCTs consistently covered across the systematically reviewed reviews or drug development with focus on bipolar depression [59] could therefore be retrieved in the respective references provided above.

### 3.2. Chemistry

**Figure 3:**
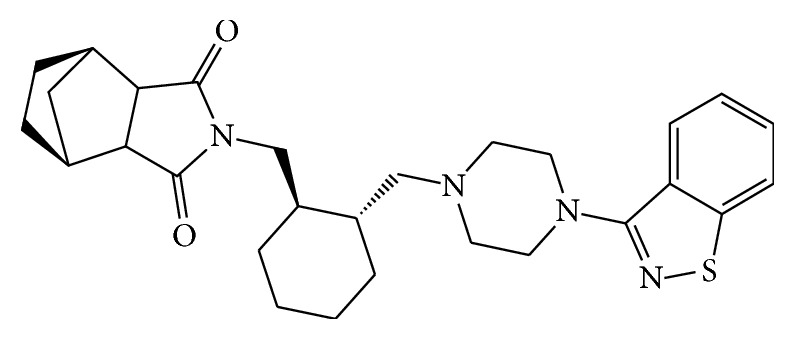
2D conformer of lurasidone (compound: CID 11237860). Additional reference at https://pubchem.ncbi.nlm.nih.gov/compound/Lurasidone_HCl#section=2D-Structure.

Lurasidone hydrochloride (molecular formula C_28_H_37_ClN_4_O_2_S) is a benzisothiazolinone derivative. Its molecular weight is 529.14 g/mol.  Figure 3 shows lurasidone chemical structure. Please access https://pubchem.ncbi.nlm.nih.gov/compound/Lurasidone_HCl#section=Molecular-Formula for additional details.

### 3.3. Essential Pharmacokinetics and Pharmacodynamics Signature

Substantial consensus exists about the pharmacological signature of lurasidone hydrochloride across sources assessing either preclinical or clinical studies on the matter [60–64], as briefly synthesized below.

#### 3.3.1. Essential Pharmacokinetics of Lurasidone

Lurasidone is metabolized by the liver via cytochrome (CYP) 3A4 [65] and it is best absorbed with 350 Kcal of food as detailed elsewhere [66]. Although the requirement to take lurasidone with food is minimal inconvenience [67], the ability to take it just once a day is an advance over alternative medications or polypharmacy regimens [63].

According to the US package insert, rifampin, a potent CYP3A4-inducer, decreased lurasidone area under the curve fivefold [68]. There are no published studies of potent antiepileptic drugs CYP3A4 inducers that are expected to dramatically increase lurasidone metabolism, including those seldom prescribed to BD patients too [69]. Nonetheless, it is possible that in high doses, oxcarbazepine or topiramate may influence lurasidone levels [70].

When prescribed as oral monotherapy for depression associated with BD-I in adults, initial dose should be 20 mg/day, with no titration needed and maximum dose being 120 mg/day depending on patient response, tolerability, and pharmacokinetics issues (in contrast to usually higher dose range of 80–120 mg/day often required for adult cases of schizophrenia, when used as monotherapy in the absence of major pharmacokinetic interactions) [61, 71, 72].

#### 3.3.2. Essential Pharmacodynamics of Lurasidone

Lurasidone is a full antagonist at dopamine (DA) D2 and serotonin (or 5-hydroxytryptophan [5-HT]) 2A (5-HT2A) receptors, with binding affinities (Ki) of 0.47 nanomole (nM) and 0.994 nM, respectively [61, 73]. However, lurasidone also has high affinity for serotonin 5-HT7 receptors (0.495 nM; comparable to D2 and 5-HT2A receptors) and has affinity as partial agonist at 5-HT1A receptors with a Ki of 6.38 nM [72]. This may be of potential interest because of preclinical findings of a possible procognitive effect mediated by action at the serotonin 5-HT7 receptor [60, 61]. Notably, 5-HT7 blockade has been postulated to boost 5-HT1A modulation by lurasidone antagonism elsewhere postulated as a reinforcement of 5-HT1A activity [74]. Lurasidone is also low- to moderate D3 antagonist [75].

Lurasidone preclinical profile was found predictive of antipsychotic, antimanic, antidepressant, and procognitive effects [61], as well as efficacy against negative symptoms of schizophrenia [63]. Moreover, its lack of affinity for some receptors (e.g., histamine H1, acetylcholine M1) would predict improved (metabolic and cognitive) tolerability with respect to alternative SGAs options approved by the FDA for BD in adults [61, 63]. Favorable D2/5-HT2A balance would predict lower propensity for extrapyramidal symptoms [64].

According to the original model proposed by Fountoulakis et al. [76], the stronger predictors for antidepressant efficacy in bipolar depression would encompass norepinephrine alpha-1, dopamine (DA) D1, and histamine antagonism, followed by 5-HT2A, by muscarinic and dopaminergic D2 and D3 antagonism, and eventually by norepinephrine reuptake inhibition and 5-HT1A agonist effect [76].

Yet, it is worth noting that the model would therefore outline a complex interaction between the major neurotransmitter systems without a single target being either necessary or sufficient to elicit the antidepressant effect in bipolar depression [76].

While serotonin reuptake inhibition may not play per se a significant role in bipolar depression [77], it is worth noticing that norepinephrine activity seems to be of great of importance [61]. Thus, early bipolar antidepressant activity excreted by lurasidone has been correlated with disinhibitory norepinephrinergic effects of agonistic activity at 5-HT1A (which may play a core mechanism in bipolar depression [78] beyond major depressive disorder [79]) and antagonism at alpha-1 norepinephrine and serotonin 5-HT2A receptors, though the presence of norepinephrine reuptake inhibition (lurasidone is also weak alpha-2a (autoinhibitory, presynaptic) antagonist (Ki = 10.8 nM) for lurasidone [64]) is essential in order to sustain it [61], whereas dopaminergic activity overall may likewise play a major role [80].

Finally, while lurasidone pharmacodynamic signature would represent a good fit of the model proposed by Fountoulakis et al. (2012) [76], additional peculiar pharmacodynamics of lurasidone [61], including strong 5-HT7 (and D2) antagonism, otherwise suggestive of procognitive and mood-enhancing activity possibly via dopamine efflux too [81–83], and negligible or null 5-HT3 and 5-HT2C interactions, otherwise accounted as major potential players for antidepressant activity in BD [84–86], could not fit the model, possibly due to lack of additional evidence on the matter (publication bias) rather than actual negligence of their neurobiological value on the matter [61].

### 3.4. Adverse Effects Consistently Documented across Varying Systematic Reviews Based on Original Studies

An updated synthesis on major and/or most common AEs with lurasidone (even) in the treatment of BD is provided elsewhere [64]. Briefly, the most commonly reported AEs were nausea, akathisia, EPS, and sedation. Negligible effects were seen with respect to changes in fasting glucose, total cholesterol, low-density lipoprotein cholesterol, prolactin elevation, and triglycerides' levels, at least in contrast with alternative SGAs and/or higher average dose regimens used for schizophrenia patients [87]. No electrocardiogram abnormalities, not even significant increase in QT interval length, have been documented in comparison with placebo or active competitor(s) from the SGA class. Stating the lack of histamine H1 and muscarinic M1, as well as strong norepinephrine alpha-1 antagonist, activities, lurasidone has also lower propensity to induce somnolence compared to most of the available SGA alternatives [88]. On the contrary, akathisia and dystonia were important, relatively common, AEs. In addition, it is worth noting that while the FDA black box warning about the risk of death in elderly patients with dementia has been issued as a class black box warning, lurasidone database does not have studies involving subjects with dementia and has no reported deaths in clinical trials in this population. Nonetheless, we purposely avoided providing quantitative synthesis of AEs herein as evidence from RCTs studies involving adult cases of BD is tentative or otherwise hampered by strong publication bias (and selection bias of included samples), and cumulative reports derived by some systematic reviews merged data from schizophrenia cases controlled as well as noncontrolled reports, with this later cases known to be prone to higher neurological side effects and being usually exposed to higher dose of antipsychotic(s) on average [64].

## 4. Discussion

The average quality of the included systematic reviews ranged between moderate-to-high scoring, as the mode (“*most common statistical presentation*”) was equal to grade letter = “A” (namely, for 8 out 12 included studies). Overall, concordance exists about the intriguing efficacy, safety, and tolerability profile of lurasidone even in the treatment of acute depressive episodes associated with BD-I, especially when indirectly compared with other FDA-approved SGAs to date.

This later issue has major implications for the policy-makers, the clinicians, the patients, and their caregivers, especially considering that most BD patients require long-lasting (virtually lifetime enduring) pharmacological treatments (ideally integrated by alternative treatment modalities), thus being particularly vulnerable to many of the common AEs documented with some of the SGA class compounds already released, especially from cognitive and cardiometabolic standpoints [89]. Thus, there is interest towards lurasidone as a safer and more tolerable, yet efficacious, alternative to current pharmacological armamentarium.

**Figure 4:**
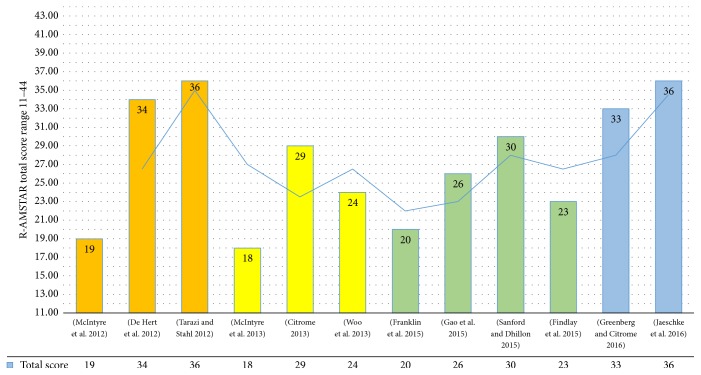
Number of studies and quality as assessed by the “Revised-A measurement tool to assess the methodological quality of systematic reviews” (R-AMSTAR) [44] total score for systematic reviews, per year. Note: 2016 records could be updated till late October.

Nonetheless, it must be remarked that the R-AMSTAR grading system is relative to the set at review, which does not necessarily mean absolute high quality of reporting for a given review out of the set at overview. This further solicits additional primary research studies (namely, RCTs to fill the gap of publication bias and allow meta-analysis of the evidence), as well as the need for higher quality of reporting of the forthcoming reviews (especially regarding the details documenting research protocols procedures and the biases encountered in the assessment of primary research, namely, publication and sponsorship bias, to this end). On the other side, the trend of publication of reviews about lurasidone confirms the clinicians' interest about this compound, even in the treatment of BD, as depicted in Figure 4.

Specifically, despite growing body of literature detailing the efficacy and tolerability of lurasidone, a complementary body of literature documenting its efficacy for the treatment of BD-I is comparatively less than the one concerning other SGAs, essentially due to the short time since initial approval (subsequently extended beyond the sole treatment of schizophrenia). This later issue may also concur the explanation why most of the “systematic” reviews on the matter essentially focused on the pharmacodynamics and/or pharmacokinetics of the drug rather than on RCT studies [90]. In this regard, it is also worth noticing that the upcoming clinical trials should ideally investigate the impact of current and/or lifetime mixed features as primary outcomes. Similarly, greater attention and stratification of the reported results by upcoming RCTs should focus on psychotic features and (emerging) suicidality, which would on turn demand for extended longitudinal follow-up, including flexible-dose regimen arms, to better investigate medication adherence to lurasidone across varying dosing patterns [91]. This is particularly true considering that BD and mood disorders in general should be better evaluated from an extended longitudinal perspective, as originally postulated by Emil Kraepelin for “manic-depressive illness” [17, 18].

### 4.1. Study Limitations

Sponsorship, publication biases, and shortage of grey literature on the matter may have limited the availability of negative result trials documented either by primary or secondary research reports. Though assessed for methodological quality, additional biases inherent to the original field trials (e.g., selection bias) may have hampered the generalizability of the overall conclusions drawn herein. Both the psychometric properties and scoring guidance of the adopted R-AMSTAR may still lack in terms of validity (measurement bias). In addition, most of the included studies were written in the English language (potential language bias). English-written papers are likely to be published more rapidly (time-lag bias) and cited more often (citation bias). This is compelling for novel compounds as the mentioned publication bias may represent an issue especially in the absence of negative results reports and in the presence of sponsored RCTs only to date.

### 4.2. Lurasidone in the Treatment of Bipolar Depression: Major Clinical Implications and Recommendations for Future Clinical Studies to “Meet the Unmet Needs”

The Program to Evaluate the Antidepressant Impact of Lurasidone (PREVAIL) planned in 2009 by lurasidone manufacturer with the goal to evaluate the efficacy and tolerability of lurasidone as both a monotherapy and as an adjunctive therapy in adult patients purportedly excluded psychotic cases of depression associated with BD-I [59]. While this later strategy was intended to ensure improvement of depressive ratings due to specific antidepressant efficacy of lurasidone rather than effect of the psychotic features (excluded per protocol), concerns regarding the chance of Berkson's bias for subsequent efficacy results are documented in the developed acute and open-phase extension trials which lead to FDA approval. Thus, future trials should ideally include representative samples of BD-I depression with concurrent psychotic features too [92] and stratify results accordingly, as alternative post hoc analyses (e.g., [59, 67, 93]) from the same original RCTs adopted stratification of reporting of the results either for patients aged 55 years or older (Dols, et al.), history of treatment resistant depression [29, 30] and/or mixed features [94]. Moreover, lurasidone loose D2 postsynaptic occupancy (fast dissociation time) compared to alternative antipsychotics may represent itself a plus in the management of psychotic features associated with core mood disturbances as well [95].

Interest about lurasidone in the treatment of depression with mixed features, even when associated with sub- [94] or full-threshold bipolarity rather than major depression [96, 97], is also a matter of vivid clinical debate, although the actual differential diagnostic role of “irritability” remains an argument of debate against current DSM-5 approach [10, 84, 98].

Despite the need for the future assessment of the above specific features and/or special populations, as outlined by most of the reviewed systematic reviews and additional commentaries on the matter, overall evidence of efficacy of lurasidone as (acute antidepressant) monotherapy for adult patients with BD has an additional translational value [20, 21], as many individuals with BD are treated with polypharmacy even in circumstances where the most adequate initial approach would be monotherapy [17, 18], with potential detrimental effects on overall treatment adherence [99, 100].

Additional outcomes in cognitive data analyses are nonetheless awaited to determine if there is a key difference between lurasidone and other SGAs with respect to efficacy [63, 101], stating both the core relevance of cognitive symptoms of bipolar depression [102, 103] and the recommendations for future trials to account for personalized or individualized medicine (including biomarkers) even in BD [104].

Among others, clinical concerns raised upon overview of qualitative and quantitative evidence of lurasidone in BD would regard the need for inclusion of active compound alternative arms in future RCTs (e.g., fixed versus standard dose head-to-head comparisons of lurasidone against OFC, quetiapine, or alternative SGAs). Once again, long-term maintenance double-blind (extension) studies are likewise warranted [105]. Ideally, independent controlled trials would test whether any significant statistical and/or clinical threshold effect would be detected in RCTs directly comparing mono- versus adjunctive therapy regimens (regardless the presence of a third arm including placebo).

The need for additional information on special populations and/or clinical presentations is likewise compelling, especially considering that trials based on the Diagnostic and Statistical Manual Fifth Edition (DSM-5) [106] specifiers of mixed features or rapid-cycling specifier are missing (or yet to be disclosed to the public at writing time), as well as additional information about otherwise relevant course and specifiers not (yet?) accounted by the DSM, including, but not limited to, predominant polarity index [53], stratification of results and specific analyses about gender, and differential comorbidity profile, which may in turn play a differential impact on both efficacy and tolerability outcome measures as recently stressed out by comprehensive evidence-based guidelines for treating BD [107].

### 4.3. Concluding Remarks and Future Perspectives

Overall, the present overview outlined concordant unmet needs and recommendations about the use and potential future avenues of lurasidone in the treatment of bipolar depression in adults, with a special emphasis towards its therapeutic potential for cases of treatment resistant and/or mixed features of bipolar depression. In conclusion, lurasidone holds clinical potential as a novel, efficacious pharmacological treatment for bipolar depression [66]. However, the present overview confirms that current data on its use in the BD-I population are limited by publication bias and standardization of trial studies design. To this end, while its monotherapy and once a day administration coupled with relatively favorable metabolic and cognitive profile indeed represent an intriguing opportunity for policy-makers and budget holders as well the prescribing clinicians and the patients, more extensive research, both longer in duration and independently conducted, is needed.

## Figures and Tables

**Table 1:** Search strategy across alternative databases (queries run on October 14, 2016).

Set	Medline (via PubMed)
1	#1 *Lurasidone Hydrochloride* [MeSH Major Topic]^*∗*^
2	**#*2 review[Publication Type] OR meta-analysis[Publication Type]***

3	Sets 1-2 were combined with “OR”

4	Bipolar disorder
5	Depression

6	Sets 1–5 were combined with “OR” & “AND”

***Most permissive (generic PubMed - no MeSH headings***)	**((Lurasidone) AND Bipolar) AND review[Publication Type]**

	**Embase**

***Most permissive***	***#1 “lurasidone”/exp OR lurasidone AND “bipolar depression”:ab,ti AND review:it***

	**Scopus**

***Most permissive***	***(TITLE-ABS-KEY (lurasidone) AND TITLE-ABS-KEY (bipolar disorder)) AND DOCTYPE (re)***

	**Cochrane Library**

***Most permissive***	***“Lurasidone in Title, Abstract, Keywords and ‘bipolar disorder' in Title, Abstract, Keywords and ‘review' in Publication Type ”***

*Note*. Words written in *italic* were used as MeSH headings; the others were used as free text.

^*∗*^Proposed entry terms, then accounted as free text integrations:

Hydrochloride, Lurasidone

Lurasidone HCl

HCl, Lurasidone

SM 13496

13496, SM

SM13496

SM-13496

SM-13,496

SM 13,496

SM13,496

Lurasidone

N-(2-(4-(1,2-benzisothiazol-3-yl)-1-piperazinylmethyl)-1-cyclohexylmethyl)-2,3 bicyclo(2.2.1)heptanedicarboximide

Latuda.

**Table 2:** Essential overview of the results. Overall, higher scores and letter grades would indicate more reliable content and related conclusions/perspectives/recommendations for future studies and unmet needs to be addressed. Scoring method detailed elsewhere [44].

Author, year of publication	Country of origin (leading author)	Information about the sample or coved original studies if multiple data sources	*N* of covered original (RCT studies, other design)	Results drawn based on the accounted evidence/adopted procedures for revision. Main conclusions and perspectives recommended by the authors	Major biases (e.g., sponsorship bias) detected according to the R- AMSTAR methodology	Score of each 1 to 11 item assessed by R-AMSTAR	R-AMSTAR TOTAL SCORE (raw)	Rank (grade letter) based on percentile of the given study
(McIntyre et al., 2013)	Canada	BD-I depression; monotherapy: acute phase	1 RCT [23]	FDA-approved agents should be prioritized over not (yet?) approved options. […“ Lurasidone has minimal propensity to weight gain and appears metabolically neutral, which would will be a significant advantage.”]	Although systematic in nature, most of the core procedures recommended for systematic review were not documented or implemented. Covered SGAs, including lurasidone, focused only on RCT leading to FDA approval, raising concerns for selection and publication biases	I = 1 II = 1 III = 2 IV = 2 V = 1 VI = 1 VII = 3 VIII = 3 IX = 1 X = 1 XI = 2	Total score = 18	Percentile in the present set = 8.3 Quartile-derived grade letter = D

(McIntyre et al., 2012)	Canada	The review focused on the pharmacodynamics and pharmacokinetics of lurasidone rather than original RCTs	Studies involving bipolar cases were underway at time of writing	Lurasidone would introduce simplicity of use and favorable metabolic advantages relative to several other SGAs. Outcomes in cognitive data analyses are awaited to determine if there is a key difference between lurasidone and other SGAs with respect to efficacy	Publication bias with respect to RCT trials (indeed, beyond the scopes of the review)	I = 1 II = 1 III = 2 IV = 2 V = 1 VI = 3 VII = 2 VIII = 3 IX = 2 X = 1 XI = 1	Total score = 19	Percentile in the present set = 16.7 Quartile-derived grade letter = D

(Franklin et al. 2015)	USA	The review focused on the pharmacodynamics and pharmacokinetics	Two acute phase trials (mono- and adjunctive therapy, resp.) [23, 24]	Lurasidone acts as high affinity D2 and 5-HT2A antagonist. Lurasidone, however, has somehow uniquely bind with high affinity with 5-HT7 compared to other SGAs. Lurasidone also acts as 5-HT1A partial agonist. Lurasidone is best absorbed with 350 Kcal of food and is metabolized by CYP34A	Lack of assessment of maintenance preliminary data (publication bias)	I = 1 II = 1 III = 1 IV = 1 V = 1 VI = 3 VII = 2 VIII = 3 IX = 2 X = 2 XI = 3	Total score = 20	Percentile in the present set = 25 Quartile-derived grade letter = C

(Citrome 2013)	USA	The review is well-grounded with regard do evidence-based methodology and cover a broad number of SGA drugs. The review focuses on the pharmacology, efficacy and tolerability profile of lurasidone and other SGAs	Two acute phase trials (mono- and adjunctive therapy resp.) [23, 24].	Although encouraging, the NNT and NNH computed about lurasidone for the acute treatment of BD-I would need additional primary research data to pool in order to allow more firm conclusions	Search strategy and selection of the not documented in full. This is with special reference to PICO/PIPO research questions	I = 1 II = 2 III = 4 IV = 2 V = 2 VI = 3 VII = 3 VIII = 3 IX = 3 X = 3 XI = 3	Total score = 29	Percentile in the present set = 58.3 Quartile-derived grade letter = A

(De Hert et al., 2012) [46]	Belgium	The review focuses on the effects of lurasidone (and asenapine, iloperidone and paliperidone) on body weight and metabolic adverse effects in both schizophrenia and BD patients	No RCT leading to FDA approval (extension) covered in the present piece of work	Based on the evidence available at writing time, lurasidone metabolic effects would resemble those of aripiprazole, amisulpride and ziprasidone closer than the other SGAs assessed in the review at issue. Additional studies are nonetheless warranted	Publication bias with respect to RCT trials (indeed, beyond the scopes of the review)	I = 3 II = 3 III = 4 IV = 2 V = 4 VI = 3 VII = 3 VIII = 2 IX = 4 X = 3 XI = 4	Total score = 34	Percentile in the present set = 83.3 Quartile-derived grade letter = A

(Greenberg and Citrome, 2016)	USA	The review focused on the pharmacodynamics and pharmacokinetics	No RCT leading to FDA approval (extension) covered in the present piece of work	The review provides a very comprehensive and updated synthesis of lurasidone pharmacological properties. Distinguishing features of lurasidone against alternative SGA are also outlined, with a special emphasis towards the very potent 5-HT7 activity excreted by lurasidone and its complex interactions with 5-HT1A partial agonist activity	Publication bias with respect to RCT trials (indeed, beyond the scopes of the review)	I = 1 II = 2 III = 4 IV = 3 V = 2 VI = 4 VII = 3 VIII = 4 IX = 4 X = 3 XI = 3	Total score = 33	Percentile in the present set = 75 Quartile-derived grade letter = A

(Tarazi and Stahl, 2012) [47]	USA	The review covers the preclinical and clinical data available about lurasidone, asenapine and iloperidone until writing time	No RCT leading to FDA approval (extension) covered in the present piece of work	The review provides an expert opinion perspective about titration, dosing and management of most common side effects eventually experienced by patients with BD in receipt of lurasidone. This allows making inference about some of the clinical unmet needs to be address by forthcoming studies	Publication bias	I = 1 II = 3 III = 4 IV = 2 V = 4 VI = 3 VII = 3 VIII = 4 IX = 3 X = 3 XI = 4	Total score = 36	Percentile in the present set = 100 Quartile-derived grade letter = A

(Gao et al., 2015) [48]	USA	The review focuses on the NNT and NNH computed for the essential SGAs approved by the FDA for the treatment of acute bipolar depression in adults, including lurasidone	Two acute phase trials (mono- and adjunctive therapy resp.) [23, 24]	Staining the relatively favorable profile of lurasidone, the authors recommend that the selection of an FDA-approved SGAs for bipolar depression should be based upon priority given to safety and tolerability issues	Publication bias	I = 1 II = 2 III = 3 IV = 2 V = 2 VI = 3 VII = 4 VIII = 2 IX = 3 X = 2 XI = 4	Total score = 26	Percentile in the present set = 50 Quartile-derived grade letter = A

(Sanford and Dhillon, 2015) [49]	New Zealand	The review provides a synthesis about the use of lurasidone in adults with bipolar depression	Two acute phase trials (mono- and adjunctive therapy, resp.) [23, 24]. Maintenance open-trial is likewise critically accounted. [28]	The review stresses out the relatively favorable profile of lurasidone in terms of both acute and potentially long-term tolerability in the treatment of BD-I depression, either as mono- or as adjunctive treatment	Publication bias, tough the present review is among the most updated and concise available at writing time	I = 2 II = 2 III = 3 IV = 2 V = 3 VI = 4 VII = 2 VIII = 3 IX = 3 X = 3 XI = 3	Total score = 30	Percentile in the present set = 66.7 Quartile-derived grade letter = A

(Jaeschke et al., 2016) [50]	Poland	The present review covers both the 2016 updates about the use of lurasidone in BD and schizophrenia, including synthesis of the pharmacological profile and essential reference to both animal and human studies	Two acute phase trials (mono- and adjunctive therapy, resp.) [23, 24]. Maintenance open-trial is likewise critically accounted. [28]	This is a very accurate, yet concise, update of the evidence up to early 2016. Nonetheless, the authors failed to expand any conclusive sections about recommendations and the unmet needs for future clinical use and research development	Publication bias but no suspiciousness of sponsorship bias	I = 1 II = 3 III = 3 IV = 2 V = 4 VI = 4 VII = 3 VIII = 4 IX = 4 X = 4 XI = 4	Total score = 36	Percentile in the present set = 100 Quartile-derived grade letter = A

(Woo et al. 2013) [51]	South Korea	This is a “preliminary” review about the potential use of lurasidone in the treatment of bipolar depression associated with BD-I in adults	No RCT leading to FDA approval (extension) could be covered in the present piece of work at writing time	Despite the publication bias and intrinsic limitation of lack of evidence, the review is formulated in a critical manner, which would contribute to providing useful clinical recommendation for the actual use of the drug and its further development	Publication bias	I = 2 II = 2 III = 2 IV = 1 V = 2 VI = 3 VII = 2 VIII = 2 IX = 2 X = 2 XI = 3	Total score = 24	Percentile in the present set = 41.7 Quartile-derived grade letter = A

(Findlay et al. 2015) [52]	USA	The review focuses on the tolerability and efficacy profile of lurasidone in the treatment of BD-I depression in adults	Two acute phase trials (mono- and adjunctive therapy, resp.) [23, 24]. Maintenance open-trial is likewise critically accounted. [28]	Tough very concise and only partially “systematic” in nature, the present review provides inputs and hints about the clinical use of lurasidone which would pave the ground for further development and address of some of the unmet needs faced by the clinical prescribers and the suffering ones	Publication bias	I = 1 II = 2 III = 2 IV = 1 V = 2 VI = 3 VII = 2 VIII = 1 IX = 3 X = 3 XI = 3	Total score = 23	Percentile in the present set = 33.3 Quartile-derived grade letter = B

BD = bipolar disorder; randomized clinical trial = RCT. R-AMSTAR = revised “assessment of multiple systematic reviews.” TRBD = treatment-resistant bipolar depression; PP = predominant polarity (of mood episodes over the lifetime course of BD) [53]. FDA = Food and Drug Administration; BD = bipolar disorder; SGA = second generation antipsychotics. NNT = number needed to treat; NNH = number needed to harm. PICO/PICO research questions: population, intervention, comparison, prediction, and outcome.

**Table 3:** 

R-AMSTAR total score	Frequency	Percentile
18.00	1	8.3
19.00	1	16.7
20.00	1	25.0
23.00	1	33.3
24.00	1	41.7
26.00	1	50.0
29.00	1	58.3
30.00	1	66.7
33.00	1	75.0
34.00	1	83.3
36.00	2	100.0

*Note*. Percentiles ≤ 20.75 would fall in the bottom 25th quartile (grade letter “D”); percentile range 20.76–27.50 = lower 50th or letter C; percentile range 27.6–33.75 = upper quartile till 75th, or letter B; percentiles ≥ 33.75 would fall in the top quartile (up to 100th percentile), or letter grade “A.”
